# Creation of a synthetic indicator of quality of care as a clinical management standard in primary care

**DOI:** 10.1186/2193-1801-2-51

**Published:** 2013-02-13

**Authors:** Ermengol Coma, Manel Ferran, Leonardo Méndez, Begoña Iglesias, Francesc Fina, Manuel Medina

**Affiliations:** 1 Institut Català de la Salut (ICS), Gran Via de les Corts Catalanes, 587-589, 08007 Barcelona, Spain; 2 IDIAP Jordi Gol (Primary Health Care Research Institute), Gran Via de les Corts Catalanes, 587, 08007 Barcelona, Spain; 3 Unitat Docent de Barcelona, Institut Català de la Salut (ICS), c/ Sant Elies 42, 08006 Barcelona, Spain; 4 EAP Vilanova 3 CAP Baix a Mar, Consorci de Serveis a les Persones, Plaça Boleranys, 5, 08800 Vilanova i la Geltrú, Spain

**Keywords:** Health care quality indicators, Primary care, Feedback

## Abstract

**Introduction:**

The development of electronic medical records has allowed the creation of new quality indicators in healthcare. Among them, synthetic indicators facilitate global interpretation of results and comparisons between professionals.

**Methods:**

A healthcare quality standard (EQA, the Catalan acronym for *Estàndard de Qualitat Assistencial*) was constructed to serve as a synthetic indicator to measure the quality of care provided by primary care professionals in Catalonia (Spain). The project phases were to establish the reference population; select health problems to be included; define, select and deliberate about subindicators; and construct and publish the EQA.

**Results:**

Construction of the EQA involved 107 healthcare professionals, and 91 health problems were included. In addition, 133 experts were consulted, who proposed a total of 339 indicators. After systematic paired comparison, 61 indicators were selected to create the synthetic indicator. The EQA is now calculated on a monthly basis for more than 8000 healthcare professionals using an automated process that extracts data from electronic medical records; results are published on a follow-up website. Along with the use of the online EQA results tool, there has been an ongoing improvement in most of the quality of care indicators.

**Conclusions:**

Creation of the EQA has proven to be useful for the measurement of the quality of care of primary care services. Also an improvement trend over 5 years is shown across most of the measured indicators.

**Electronic supplementary material:**

The online version of this article (doi:10.1186/2193-1801-2-51) contains supplementary material, which is available to authorized users.

## Background

One of the priorities of health systems is ensuring the quality of primary care (Nietert et al. 2007). To achieve this objective, multiple indicators have been developed to assess the quality of clinical practice (McColl et al. 1998). Nonetheless, utilization of numerous partial indicators makes it difficult to achieve a global interpretation and to compare individuals as well as groups or centres. In many situations, the use of fewer but more comprehensive indicators is preferable. Along this line, some composite, or synthetic, indicators have been defined that condense the measurement of several subindicators into one value, thereby facilitating interpretation and better representing the process of healthcare delivery (Saturno 2004). The possibility of summarizing quality of care in one measurement has made these synthetic indicators very attractive for comparisons between different centres and professionals. Among other characteristics, good health indicators should be valid, evidence-based, comparable, modifiable, directed toward health problems of importance due to their severity or frequency in primary care patients, feasible to obtain without overburdening the healthcare professional and finally, they must deliver health benefits to the population with minimal secondary effects (Brown and Lilford 2006; NHS Institute for Innovation and Improvement). One of the circumstances that has especially favoured this process of constructing synthetic indicators has been the development of electronic medical records and the availability of tools to manage large databases.

In recent years there has also been increasing international interest in “pay for performance” programmes for individual health professionals (Gérvas and Pérez 2008) and in the development of evidence-based synthetic indicators to objectively measure clinical performance. In 1994 this new payment method began in Australia (Medicare Australia) and has been extended to Canada (Pink et al. 2006), the United States (Rosenthal et al. 2005), New Zealand (Perera et al. 2007) and the United Kingdom (Roland 2004; Doran et al. 2006), among others (Gérvas and Pérez 2008; Doran et al. 2008; Engels et al. 2005). Some of these systems have incorporated several different models of clinical indicators. The most well-established effort is that of the National Health Services (NHS) of the United Kingdom, which in 2004 developed the Quality Outcomes Framework (QOF), a payment system for primary care physicians. This system facilitates a single quality measurement that distributes 1000 points across four domains (Roland 2004; Doran et al. 2006; Lester and Majeed 2008). In the United States, there are also systems of clinical indicators that measure outpatient care (Rosenthal et al. 2005; The Ambulatory Care Quality Alliance Recommended Starter Set). One of these systems is the Summary Quality Index (SQUID), a composite measure of the quality of primary care that consists of 36 quality indicators related to a variety of fields and supported by a solid scientific base (Nietert et al. 2007). As has occurred in other environments, utilization of electronic medical records in primary care is widespread in Spain, but there is no global system to describe the clinical performance of healthcare professionals and centres. However, at a more local level, very useful and well-structured efforts have been observed, such as that in Tarragona province (Catalonia) which selected 10 health problems in primary care that became a standardized 17-item scale of healthcare quality (Vila et al. 2006).

The Catalan Health Institute (*Institut Català de la Salut*, ICS) is a public enterprise of the Generalitat de Catalunya, the Catalan government. It is the primary provider of primary care services and covers approximately 80% of the population (~5 800 000 patients). Beginning in 2005, a series of circumstances combined to encourage administrators to pay greater attention to measuring clinical quality. First, the process of computerizing medical records was practically completed. All primary care practices now routinely enter clinical data in a software application called e-CAP (*Estació Clínica d’Atenció Primària*), the electronic medical records system for primary care in Catalonia. Secondly, there was a growing effort to incentivize healthcare professionals within a framework of variable, evidence-based compensation known as “pay for performance” (Cortés Pérez et al. 2009; Iglesias Pérez et al. 2008). This led to a need for a standard, homogeneous quality measure for health professionals who provide patient care (Fina Avilés et al. 2010). Initially, then, the established priority was to design a synthetic indicator to base healthcare on the best available scientific knowledge and focus attention on the patient; eventually the indicator also became a tool in “pay for performance” determinations for doctors and nurses (Peiró 2004).

This article describes the construction and shows the results of the healthcare quality standard known as the EQA (*Estàndard de Qualitat Assistencial*, the Catalan acronym for Healthcare Quality Standard), a synthetic indicator designed to measure the quality of care provided by primary health care professionals and serve as a useful tool to improve clinical practice.

## Methods

The EQA is a synthetic indicator that combines distinct clinical subindicators. It was designed between May and December 2006 and the objective was to obtain an indicator that would measure quality of care provided to a population assigned to a primary care team (PCT). Later, in 2007, a series of changes in methodology were implemented and these have remained in effect. The subindicators comprising the EQA are calculated by the Information System for Primary Care Services (SISAP), which draws information directly from the e-CAP software.

The methodology for the construction of the EQA included: 1) Establishment of the reference population and the theoretical basis for the standards; 2) Selection of health problems to be included in the EQA; 3) Definition of subindicators; 4) Selection of subindicators; 5) Deliberation about the subindicators and final construction of the EQA; and 6) Publication of EQA results.

### Establishment of the reference population and theoretical principles

Initially, the reference population for the EQA was the population assigned to a PCT. Later, this population was modified and beginning in 2008 was defined as the population assigned to and receiving attention from a PCT at least once in the preceding three years for any reason. Each subindicator included only those patients who met specific criteria. For example, a subindicator that determines the proportion of patients treated with beta-blockers only makes sense in a restricted population such as patients with ischemic heart disease, or a subindicator that monitors glycated haemoglobin should only include patients with diabetes mellitus.

On the other hand, the theoretical principles that frame the design of the EQA were: a) To encourage quaternary prevention and avoid overtreatment; b) To provide global information about clinical practice in primary care; c) To combine the best available tests with professional judgment in defining the subindicators; and d) To guarantee the quality of the subindicators and the feasibility of collecting them from the electronic medical record.

### Selection of the health problems

Construction of the subindicators that comprise the EQA was based on a quantitative analysis of the health problems most frequently treated in primary care, both in the population older than 14 years and in the paediatric population. This included an analysis of the diagnoses codified in the visits by family physicians, paediatricians or nurses at all of the ICS primary care sites from January through December of 2006 (4 680 706 users and a total of 23 327 557 visits).

### Definition of subindicators

Once the health problems had been selected, following a Delphi methodology, we asked at least two experts on each diagnosis to formulate a clinical management indicator. Instructions were sent by e-mail, along with a standard form to collect the following information for each indicator: definition of the indicator, numerator, denominator, target age group and evidence level, according to the *North of England Evidence Based Guideline Development PROJECT* (Eccles et al. 1998).

### Selection of subindicators

All of the proposed indicators underwent a structured review by two groups of evaluators based on the criteria of validity, reliability, ease of calculation and scientific evidence. The first review used a paired comparison process in which two evaluators, independently and using the established criteria, accepted or rejected the indicators. In the case of disagreement, a decision was taken by consensus of the entire group of evaluators. All of the selected indicators were then submitted to a new consensus review by a second group of evaluators. To maintain a certain stability in the calculation of indicators, an annual review process was established; immediate application of modifications would occur only if an error in design or calculation were detected or new evidence appears that justifies such a change.

### Deliberation about subindicators and final construction of the EQA

After selecting the indicators, a new revision was made with the following objectives: to group similar indicators; to link indicators to health problems and to remove prevalence indicators because all indicators were adjusted by detection.

Evaluators also assigned the appropriate weight that each of them would have in the final computation of the EQA. This deliberation involved a multidisciplinary group of primary care professionals, who based their consideration on previously defined criteria of severity (0 to10 points), magnitude (0–10), impact of primary care (0–5) and effectiveness (0–5) (NCMC: non-compensatory multi-criteria approach).

Finally, the EQA was constructed as a synthetic indicator that assigns a global score on a scale of 0 to 1000 points, i.e., the sum of the scores obtained on the subindicators comprising the standard. All of the subindicators are calculated to produce a global score for each PCT and most of them are also calculated for each professional. Whenever possible, the indicators combine the quality of care and disease detection in one sole measure (see Additional file 1 for more details about EQA calculation). For each subindicator, a minimum value was also established at the beginning of 2007, corresponding with the 20^th^ percentile of the distribution. Below this value, 0 points are assigned on that indicator. Similarly, an optimum value is set that corresponds with the 80^th^ percentile of the distribution, beyond which the maximum value for that indicator is assigned. Between these two values, a score between 0.1% and 99.9% of the maximum possible score was determined. The score for the indicator resulted from the sum of all the partial scores assigned to each of its subindicators. Therefore, the results of the subindicators comprising the EQA are now compared with minimum and maximum targets established in January of each year, based on the 20^th^ and 80^th^ percentiles of the results over the previous 12 months. In addition, each primary care professional or team has an individual target on the synthetic indicator that is drawn from the baseline score and rewards efforts to improve.

### Publication of the EQA results

Once the EQA was constructed, primary care professionals and teams could consult their clinical indicators from their workstations. These look-ups left a record in the information system that allowed us to determine that professionals were accessing their results to see whether they were meeting their patient care goals. Since 2007, the information has been updated monthly and results for each professional compared with his or her surroundings (e.g., for that entire PCT or the average levels and total results for all ICS PCTs) and with their baseline scores. Results are colour coded to facilitate both their interpretation and an assessment of the professionals’ response. Whenever possible, in addition to numeric scores, each professional can view a list of patients who are not meeting the established criteria for EQA indicators, with the objective being to review those cases individually.

## Results

### Construction of the EQA

For the different phases of constructing the EQA, participation included 107 healthcare professionals, 73 of whom took part in defining the subindicators and 34 in the later selection and deliberation phases and the final construction of the synthetic indicator.

#### Selection of the health problems

Health problems that represented the 80% of the demand for medical attention in primary care were selected. These ICD10 codes were gathered in 91 health problems groups according to physiological, anatomical and etiological criteria (between 1 and 9 codes per group). For more information see Additional file 2.

#### Definition of subindicators

In order to define a series of subindicators based on the selected health problems, 133 recognized experts working within the Catalan healthcare network were contacted, of whom 73 (54.9%) responded. These respondents provided a total of 339 proposed indicators.

#### Selection of subindicators

All of the 339 proposed indicators were reviewed in two steps of the process (paired comparisons and consensus review). Of these, 210 indicators were rejected, 26 were accepted as proposed and 103 were accepted with some modifications.

#### Deliberation about subindicators and final construction of the EQA

Of the 129 indicators subjected to deliberation, 25 were discarded due to feasibility problems in their calculation. After the fusion and rejection of some indicators as described in methods (prevalence indicators and indicators that measure the same) 61 indicators were selected. So, of the 91 initial health problems, 21 in the population older than 14 years had the necessary characteristics to be evaluated in a centralized manner using the electronic medical record.

Therefore, the EQA for primary care was constructed from 61 subindicators, 39 of them dealing with the population older than 14 years and 22 targeting the paediatric population. At the level of the individual professional, the calculation incorporates just 27 of the subindicators for the population older than 14 years and 16 paediatric subindicators, since the number of cases for the rest of the indicators would not permit meaningful individual EQA calculation. Table 1 contains the indicators that comprise the EQA and their weighting and Additional file 3 contains their definitions.

**Table 1:** **Structure and weighted scoring of the synthetic indicator “Healthcare Quality Standard 2007**

Clinical condition in adults		Relative weight
	Indicator	
***Ferropenic anaemia***		
1	Diagnosis of new cases	14
2	Follow-up	12
***Social assessment***		
3	Of individuals who are dependent on family members	13
4	Of frail elderly individuals	16
***Cerebrovascular disease (CVD) and/or transient ischemic attack (TIA)***		
5	Antiplatelet/anticoagulant treatment	30
6	Lipid control	26
***Ischemic heart disease (IHD)***		
7	Beta-blockers treatment	26
8	Antiplatelet/anticoagulant treatment	26
9	Lipid control	23
***Hypercholesterolemia***		
10	Cardiovascular Risk (CVR) record of hypercholesterolemia	30
***Atrial fibrillation (AF)***		
11	Antiplatelet/anticoagulant treatment	38
***Arterial hypertension (AHT)***		
12	Blood pressure (BP) control	44
13	Blood pressure (BP) control in at-risk population (IHD, DM, CVD/TIA, Chronic Kidney Failure)	25
***Heart failure (HF)***		
14	ACEI/ARB treatment	26
15	Beta-blockers treatment	23
***Chronic hepatitis C***		
16	Vaccination for hepatitis B virus	23
***Alcohol use***		
17	Screening for alcohol use	26
***Tobacco use***		
18	Screening for tobacco use in at-risk population	26
19	Smoking cessation	29
***Diabetes mellitus 2 (DM2)***		
20	Screening and onset prevention	20
21	HbA1c control	30
22	Screening and prevention of diabetic retinopathy	23
23	ACEI/ARB treatment in DM2 with chronic nephropathy	24
***Cognitive deterioration***		
24	Syndrome diagnosis, new cases of cognitive deterioration	17
25	Home health care interventions for a safe living environment for patients with dementia	13
26	Assessment of caregiver burnout	16
***Impacted earwax***		
27	Removal of earwax plugs in primary care	10
***Asthma***		
28	Diagnosis of new cases of asthma	22
***Vaccinations***		
29	Flu shots in patients aged ≥60 years	29
30	Flu shots in at-risk patients	20
31	Pneumonia shots in patients aged ≥60 years	5
32	Tetanus shots	35
***Chronic obstructive pulmonary disease (COPD)***		
33	Diagnosis of new cases	23
34	Training in use of COPD inhalers	12
***Home health care (ATDOM)***		
35	Case complexity assessment of patients with ATDOM	16
36	Assessment of pressure ulcers risk	26
***PREALT***		
37	Contact within 48 hours with PREALT patients	12
***Nephritic colic***		
38	Proper treatment of nephritic colic	10
***Prostate cancer***		
39	Avoid improper use of PSA	11
**Clinical condition in children**		**Relative weight**
	**Indicator**	
***Preventive care***		
40	Screening for congenital metabolic diseases	7
41	Introduction of foods at recommended stages	7
42	Systematic infant vaccinations (0–14 years)	10
43	Control of growth and development (0–2 years)	8
44	Screening for passive smoking ≤2-year-olds	7
45	Maintenance of maternal lactation	6
46	Measles vaccination at 13 years	6
47	Dental cavity preventive treatment (6–12 years)	7
48	Screening for ocular diseases (0–6 years)	8
49	Flu shots in at-risk children <15 years	7
50	Screening for toxic habits (11–14 years)	7
***Increasing capacity to resolve cases at primary care level***		
51	Umbilical hernia in children ≤3 years	5
52	Contagious mollusk and viral warts (0–14 years )	6
53	Neonatal dacryocystitis <9 months	6
***Acute disease***		
54	Treatment of acute gastroenteritis (3 months to 14 years )	7
55	Treatment of tonsillitis, pharyngitis or pharyngotonsillitis <3 years	7
56	Treatment of acute bronchiolitis <2 years	6
57	Treatment of catarrh in upper respiratory infection or flu <15 years	7
58	Treatment of acute nonsuppurative otitis media (2–14 years )	7
***Chronic disease***		
59	Diagnosis of childhood asthma (7 a 14 years )	8
60	Calculation of body mass index in obesity or weight gain (6–14 years )	7
***Social assessment***		
61	Social assessment of children with a disability (<15 years)	4
Total		1000

In the development of subindicators for the population older than 14 years, the clinical categories for which the most indicators were established were diabetes mellitus 2 and vaccinations, with 4 subindicators in each case; these also took on the highest weighting, with 97 and 89 points, respectively. Nonetheless, the two indicators with the highest weighting were blood pressure control in patients with arterial hypertension, with 44 points, and antiplatelet/anticoagulant treatment in patients with atrial fibrillation, with 38 points.

Between 2007 and 2010, the EQA has seen some modifications in the number of paediatric indicators and the calculation of some of these. In 2010 the paediatric portion of the EQA had 26 subindicators, 20 of which are calculated at the level of the individual professional. Some of these changes resulted from suggestions received from healthcare professionals and scientific societies. For example, in the first year of EQA implementation (2007) 142 suggestions directly or indirectly related to the EQA were received and analysed. None of the modifications performed affected the definition of subindicators.

### Evolution of the indicators

**Figure 1:**
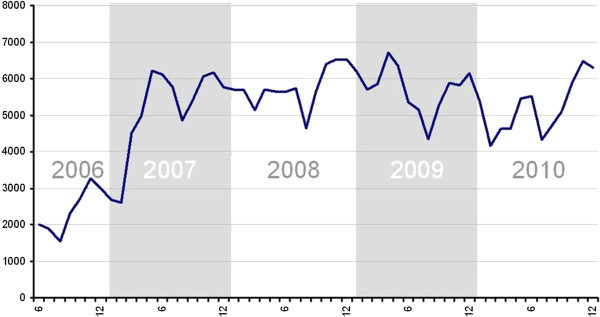
**Number of healthcare professionals who consult their EQA quality outcomes.**

The current EQA is calculated in an aggregate form for 296 PCTs, representing 4340 family physicians and paediatricians, and 4082 nurses. These professionals receive monthly information updating their results on the relevant indicators. The EQA calculation uses an automatic computerized process that extracts data from electronic medical records; the results are published in a follow-up website linked to their workstation (Coma Redon and Méndez Boo 2010). Figure 1 shows the growing trend of healthcare professionals consulting their EQA results each month using the online tool. In just four years (2006–2010), the number of professionals accessing their results increased from 1989 in June 2006 to 6305 in December 2010; since early 2007, this number has remained between 5000 and 6000 individuals, showing a slight decrease during the summer months, an increase at year-end, and a maximum peak (6701) to date in April 2009. This means that, on average, approximately 71% of all PCT professionals for whom data are published have consulted the site once a month since 2007.

**Figure 2:**
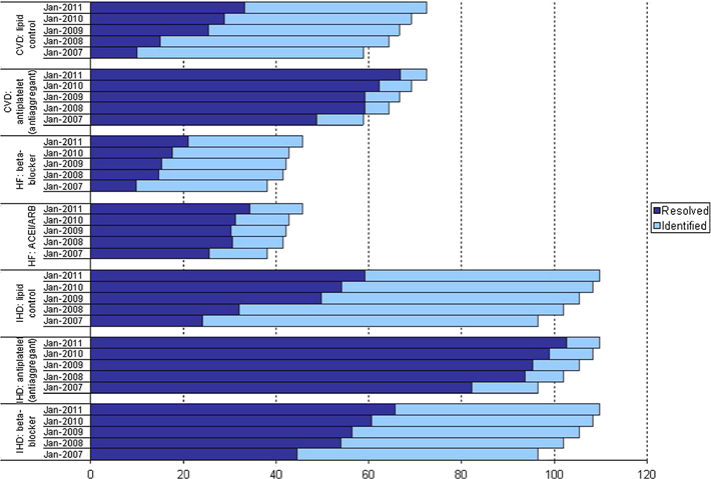
**Examples of subindicators, with numbers of patients diagnosed and managed.**

**Figure 3:**
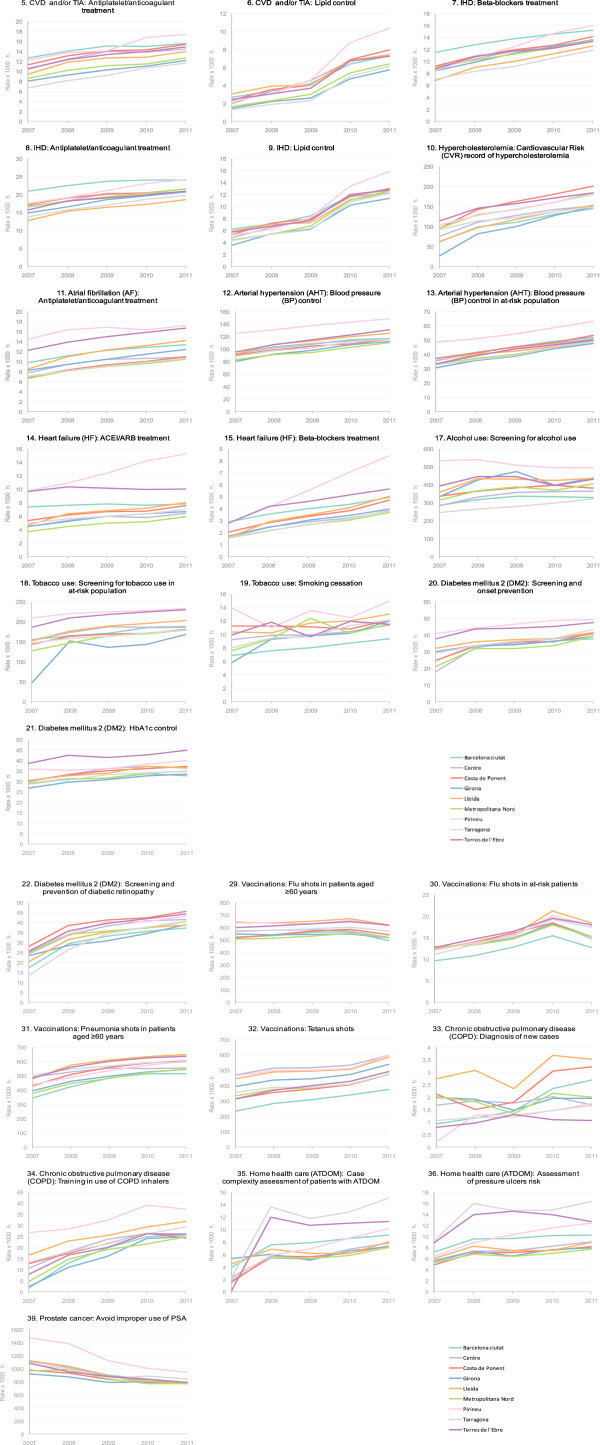
**a. Evolution of EQA subindicators**_**(5 to 21)**_**by PCT greater areas.** Resolved rates by 1000 inhabitants (h). January 2007 - January 2011. **b.** Evolution of EQA subindicators _(22 to 39)_ by PCT greater areas. Resolved rates by 1000 inhabitants (h). January 2007 - January 2011.

Together with this incremental use of the online tool to view EQA results, an ongoing improvement has been observed in most of the quality indicators. Figure 2 shows the number of patients diagnosed and, of these, how many met the criteria established for some key indicators among the years. In all cases, both the number of recorded diagnoses and the indicators being met have increased. Therefore, between January 2007 and December 2010 there was an increase of 230.78% in patients with an ischemic cerebrovascular accident who had good lipids control, of 33% in heart failure patients receiving an ACEI (angiotensin-converting-enzyme inhibitor) or ARB (angiotensin receptor blockers) prescription, of 26% in cases of ischemic heart disease receiving proper antiplatelet treatment, and of 21% in infants receiving all recommended vaccinations (Table 2). Figure 3 shows the evolution of the indicators performed at the level of the individual professional. In the better part of them, the rates of resolved cases have increased over the various years.

**Table 2:** **Difference (%) of resolved cases between 2007 and 2010 by subindicator**

INDICATOR	January 2007	December 2010	Difference	% Difference
**Ischemic heart disease**				
Treatment with beta- blockers	44,484	66,338	21,854	49.13
Treatment with antiplatelets	82,120	103,482	21,362	26.01
Control of lipids	24,269	60,105	35,836	147.66
**Heart failure**				
Treatment with ACEI or ARB	25,705	34,342	8,637	33.60
Treatment with beta- blockers	9,738	21,073	11,335	116.40
**Ischemic cerebrovascular accident**				
Treatment with antiplatelets	48,851	67,265	18,414	37.69
Control of lipids	10,127	33,498	23,371	230.78
**Childhood vaccinations**	563,524	684,125	120,601	21.40

## Discussion and conclusions

This article describes the methods used to create the EQA, a synthetic indicator of the quality of healthcare, and the results after four years of regularly calculating and reporting the results. The characteristics of the EQA are aligned with those described as essential for good indicators (Houghton and Rouse 2004; Bell and Levinson 2007): it is simple to calculate (the subindicators are proportions); easy to interpret, both for healthcare professionals who periodically look at their results and for various levels of management; relevant in the sense that the subindicators are based on health problems that stand out in primary care; and can be validated by feedback from healthcare professionals.

Among the strengths of the EQA, which it shares with other synthetic indicators such as SQUID (Nietert et al. 2007; Roland 2004), is that it is calculated automatically by a process that takes advantage of data contained in electronic medical records. This has advantages for the speed and reliability of the calculation, and decreases any potential observer bias. In addition, calculating the EQA at the patient, professional and PCT levels permits the identification of patients who are poorly controlled as well as comparisons with other groups, both of patients and of healthcare professionals and centres.

Other efforts to create a synthetic indicator of healthcare quality have been described in the literature before, such as the process used to construct the QOF or SQUID. Although it was possible to use the structure of existing indicators, a new synthetic indicator was performed because of the local acceptability and difficulties in the calculation of some subindicators with existing data. However, these three indicators share certain characteristics, being based on clinical indicators focussed on health problems selected for their prevalence or relevance in terms of impact (Roland 2004; Doran et al. 2006), working with a 12-month timeframe, or using a common algorithm (choose at-risk patients, determine whether they meet indicator, and calculate the result). In addition, the selection of criteria for the choice of the EQA subindicators (scientific evidence, availability, feasibility, etc.) is similar to that of SQUID, and many of the same pathologies were selected in both cases (arterial hypertension, HF, IHD, diabetes, COPD, vaccination, etc.). This should come as no surprise, precisely because both indicators are based on the most prevalent pathologies in primary care.

Among these common characteristics we would highlight the feedback from healthcare professionals, which has been very positive and useful. The EQA has been and remains under continuous development. Although there have been only small modifications in the definition of the indicators, there have been numerous changes in the manner of obtaining data, especially in the first and second years, as a result of user suggestions sent by e-mail. Thanks to the publication of EQA data and to being able to verify them using the patient lists provided, healthcare professionals have served as external validators, demanding changes in the information processing that collected data from the different types of records.

Despite similarities with other synthetic indicators, there are differences as well, such as the EQA weighting of each subindicator by its importance and, especially, the concept of exclusion. In the QOF the physician can exclude those patients considered irrelevant to an indicator. For example, the professional could exclude a patient with terminal cancer from an indicator of controlled cholesterol levels (Roland 2004; Doran et al. 2006). Such exclusions are intended to avoid unnecessary treatment by doctors seeking to improve their performance results. Nonetheless, this type of exclusion also could lead to manipulation of the results: the professional could improve the results on an indicator by improper exclusion of patients who did not meet that indicator.

There are several possible ways of addressing the problem of patients for whom some indicator is contraindicated. Doran et al. (2008) points out three possible solutions: to design indicators that incorporate all possible exceptions in their calculations, to permit healthcare professionals to exclude patients, or to set performance targets below 100%. The first two options have the problem of converting the indicators into something very complicated and susceptible to fraudulent exclusions. Nonetheless, an argument in favour of patient exclusions is that if the limits for the indicators are very high, some patients may be inappropriately treated because they cannot be excluded (Doran et al. 2008). On the other hand, if the limits are too low they allow doctors to achieve the maximum score without treating all eligible patients. In our case, we did not consider the possibility of allowing healthcare professionals to exclude patients in this instance and decided to set targets below 100%, combined with a calculation of expected prevalence, to ensure a minimum denominator (Additional file 1).

Of course, the risk of underreporting some pathologies in poorly controlled patients always exists. To avoid underreporting, the QOF indicators included an audit of randomly selected doctors and of some who were suspected of fraudulent results. This is an important point because the data were entered by the doctors themselves (Doran et al. 2006). In the EQA, the information was collected directly from the medical record, so there should not be many fraudulent cases. In this sense, the development of the EQA opted for a system that could control against potential manipulation and did not demand 100% compliance with the indicator. Therefore, the concept of expected prevalence was used, which allowed us to ensure a minimum prevalence for each indicator. In addition, in order to not penalize situations such as voluntary exclusion from treatment or physician decisions based on specific cases, we determined that 80% compliance (both for expected prevalence and for resolution) would receive the total score for each subindicator. As described in the results section, the number of diagnoses (and therefore of records) has increased each year, which leads us to believe that these possible exclusions are not relevant to the EQA. Nonetheless, it could be of interest to include specific, identifiable exclusions by exploiting the electronic medical record data in these cases of evident contraindications. In this sense, we have considered incorporating these exclusions in a new version of the EQA, as long as they could be assessed in a centralized fashion.

Some authors have found that improvements in indicators linked to pay for performance were due more to increased entries in the registry than to improvements in clinical practice (Bell and Levinson 2007; Petersen et al. 2006). Nonetheless, other studies indicate that economic incentives are a potent stimulus to modify professional conduct (Gené Badia 2007), although this can only be achieved if the objectives are based on records that cannot be manipulated by the healthcare professionals. Therefore, the EQA indicators combine identification of health problems, conduct of laboratory tests, control of chronic diseases, treatments prescribed, administration of vaccines, etc. These fields are difficult to manipulate because of their relevance and variety.

Monitoring the indicators can also provoke unexpected consequences such as the deselection of patients, over-treating patients without deriving any benefit and the neglect of areas not covered because of lack of information (Kerr and Fleming 2007). In this sense, a common criticism of quality indicators linked to pay for performance is that healthcare professionals will focus exclusively on these and not pay attention to other important aspects of clinical practice (Lester and Majeed 2008; Bell and Levinson 2007). Although this outcome is difficult to assess and inherent to all indicators linked to pay for performance, more studies are needed to allow us to quantify the importance of this limitation.

Finally, it is important to point out that the EQA reflects only a small part of the work of primary care professionals, that part that can be measured. Other important but non-quantitative dimensions or pathologies such as mental illness or acute pathologies are difficult to measure and are not included in the EQA. This limitation is also found in other synthetic indicators such as the QOF (Roland 2004), where the indicators represent only part of clinical practice. The underreporting of certain conditions is therefore common to all databases but need not impede our continuing efforts to improve the registry and eventually incorporate other aspects of primary care that cannot be readily evaluated with the resources available to us today.

## Electronic supplementary material

Additional file 1:: **Calculation and components of the synthetic indicator.** (DOC 74 KB)

Additional file 2: **Health problems groups, codes included and encounter rate by 1000 patients.** (XLS 18 KB)

Additional file 3:: **Definition of subindicators of the EQA.** (DOC 78 KB)

## Abbreviations

ACEI: Angiotensin-converting-enzyme inhibitor
AF: Arterial fibrillation
AHT: Arterial hypertension
ARB: Angiotensin receptor blockers
ATDOM: Home health care
BP: Blood pressure
CVD: Cerebrovascular disease
COPD: Chronic obstructive pulmonary disease
CVR: Cardiovascular risk
DM2: Diabetes mellitus 2
eCAP: Electronic health record in Catalonia
EQA: Estàndard de Qualitat Assietncial (Healthcare quality standard)
HF: Heart failure
ICD-10 code: International Classification of Diseases
ICS: Institut Català de la Salut (Catalan Institut of Health)
IHD: ischemic heart disease
NHS: National Health Services
NCMC: Non-compensatory multi-criteria approach
PCT: Primary care teams
PSA: Prostate-specific antigen
QMAS: Quality Management and Analysis System
QOF: Quality Outcomes Framework
SISAP: Sistema d’Informació dels Serveis d’Atenció Primària (Information System for Primary Care Services)
SQUID: Summary Quality Index
TIA: Transient ischemic attack.
