# Supplementary material for: Creation of a synthetic indicator of quality of care as a clinical management standard in primary care
Source: Springerplus. 2013 Feb 13;2:51. doi: 10.1186/2193-1801-2-51 (PMC3581769; doi:10.1186/2193-1801-2-51)
Supplement: Supplementary file 1 — Additional file 1:: Calculation and components of the synthetic indicator. (DOC 74 KB) [file 40064_2012_92_MOESM1_ESM.doc]

**Additional file 1 . Calculation of the synthetic indicator**

The EQA is calculated by adding the score of all the subindicators (sub_1_....sub_n_):


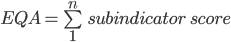
.

**Subindicator scores** take values between 0 and the relative weight. They are calculated from 4 variables:

1. The minimum value
2. The maximum value
3. The relative weights (shown in Table 1).
4. The subindicator result

The variables implicated in the score calculation are summarized and categorized in Figure 1 of this technical appendix.

**Figure 1. Summary of the components of the EQA subindicators.**


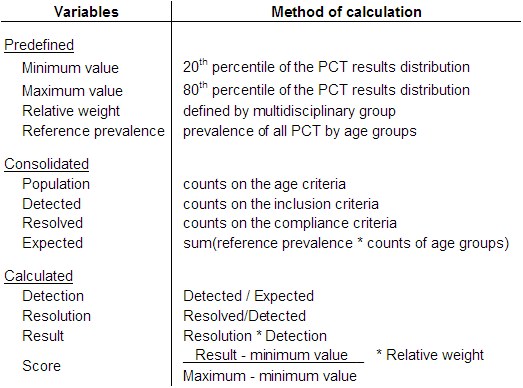


The first 3 variables used in the calculation of subindicator scores are predefined. Accordingly, they are set at the beginning of the year, their values are shared in calculations across every analysis unit (PCT, healthcare professionals) but may differ between subindicators.

**Minimum value:** corresponds with the 20^th^ percentile of the PCT results distribution. 0 score points are achieved on the subindicator if results are less than or equal to this value.

**Maximum value:** corresponds with the 80^th^ percentile of the PCT results distribution. Maximum score points equal to relative weight of the subindicator are achieved if results are greater than or equal to this value.

**Relative weight:** The score of each subindicator in the synthetic indicator. The EQA weighting of each subindicator is performed by its importance by a multidisciplinary group following a non-compensatory multi-criteria approach based on severity, magnitude, impact of primary care and effectiveness.

**Subindicator result** is best explained by breaking down its calculation method. It is the product of two main concepts: Resolution and Detection.

**Resolution:** is the proportion of compliance with the definition of the subindicator, such as the proportion of detected cases that have been resolved. For example, the resolution of indicator 21 (HbA1c control in DM2) is the proportion of DM2 patients between 15 and 79 years who have a HbA1c control <8%. According to technical appendix 3 it is also possible to define resolution as: compliance criteria / population included; or equivalently:


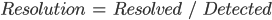


**Detection:** Proportion of actual prevalence (detected cases) compared to the expected prevalence; or equivalently:


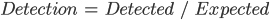


**Expected Prevalence:** The expected prevalence of a health condition in the assigned population of a PCT or a healthcare professional is obtained by applying the reference prevalence of active cases of the health condition for each age group. The reference prevalence was initially estimated on the basis of the prevalence obtained in a group of 20 PCTs with a high level of computerization and patient registration. Since 2008, when the level of computerization had improved at all of the ICS primary care sites, the expected prevalence has been calculated on the basis of the average patient age structure of all these sites.The reference prevalences are updated annually.

The remaining components are calculated simply by the adding of counts. Their definition and group relationships are showed in detail in Figure 2 of this technical appendix.

**Figure 2. Detail of the consolidated components of the EQA subindicators.**


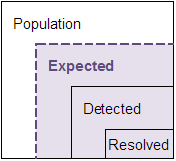


**Population**: Those in the age group required for the subindicator

**Detected**: Those in the population group with all the inclusion and none of the exclusion criteria for the subindicator; usually selected diseases or healthcare conditions, refer to appendix 3 “population included” sections.

**Resolved**: Those in the detected group in compliance with subindicator criteria, refer to appendix 3 “Compliance criteria” sections.

**Expected**: The theoretical number of detected population, once adjusted by the reference prevalence for the age group distribution of the PCT or healthcare professional.
