# Supplementary material for: Creation of a synthetic indicator of quality of care as a clinical management standard in primary care
Source: Springerplus. 2013 Feb 13;2:51. doi: 10.1186/2193-1801-2-51 (PMC3581769; doi:10.1186/2193-1801-2-51)
Supplement: Supplementary file 3 — Additional file 3:: Definition of subindicators of the EQA. (DOC 78 KB) [file 40064_2012_92_MOESM3_ESM.doc]

**Additional file 3. Definition of EQA subindicators**

**Indicator 1. Ferropenic anaemia: Diagnosis of new cases**

- Population included:
  - > 14 years.
  - New diagnosis of ferropenic anaemia (ICD10 codes: D50%).
- Compliance criteria:
  - To have recorded the hemoglobin and ferritin values at the moment of ferropenic anaemia diagnosis (between one month before and one month after diagnosis).

**Indicator 2. Ferropenic anaemia: Follow-up**

- Population included:
  - > 14 years.
  - New diagnosis of ferropenic anaemia (ICD10 codes: D50%).
- Compliance criteria:
  - Hemoglobin and ferritin values (control analysis) between 3 to 6 months after the beginning of the treatment.

**Indicator 3. Social assessment: Of individuals who are dependent on family members**

- Population included:
  - > 14 years.
  - To have registered the ICS specific code of “Individuals who depend on family members”.
- Compliance criteria:
  - To have a social assessment in the last two years.

**Indicator 4. Social assessment: Of frail elderly individuals**

- Population included:
  - >79 years.
  - To have the ICS specific code of “problems related with people living alone”
- Compliance criteria:
  - To have a social assessment in the last two years.

**Indicator 5. CVD and/or TIA: Antiplatelet/anticoagulant treatment**

- Population included:
  - >14 years.
  - Diagnosis of cerebrovascular disease and/or transient ischemic attack (ICD10 codes: G45%, G46%, I63%, I64, I67.8, I67.9, I69, I69.3,I69.4,I69.8).
- Compliance criteria:
  - At least one active treatment of:
    - Vitamin K antagonist.
    - Platelet aggregation inhibitors (not heparin).
    - Aspirin.

**Indicator 6. CVD and/or TIA: Lipid control**

- Population included:
  - >14 years.
  - Diagnosis of cerebrovascular disease and/or transient ischemic attack (ICD10 codes: G45%, G46%, I63%, I64, I67.8, I67.9, I69, I69.3,I69.4,I69.8)
- Compliance criteria:
  - At least one cLDL determination in the last year.
  - Last value of lipid control (cLDL) < 120 mg/dl.

**Indicator 7. IHD: Beta-blockers treatment**

- Population included:
  - >14 years.
  - Diagnosis of ischemic heart disease (ICD10 codes: I20%, I21%, I22%, I23%, I24%, I25%).
- Exclusion criteria:
  - Atrioventricular block (second and third degree).
- Compliance criteria:
  - At least one active treatment of beta-blockers.

**Indicator 8. IHD: Antiplatelet/anticoagulant treatment**

- Population included:
  - >14 years.
  - Diagnosis of ischemic heart disease (ICD10 codes: I20%, I21%, I22%, I23%, I24%, I25%).
- Compliance criteria:
  - At least one active treatment of:
    - Vitamin K antagonist.
    - Platelet aggregation inhibitors (not heparin).
    - Aspirin.

**Indicator 9. IHD: Lipid control**

- Population included:
  - >14 years.
  - Diagnosis of ischemic heart disease (ICD10 codes: I20%, I21%, I22%, I23%, I24%, I25%).
- Compliance criteria:
  - At least one cLDL determination in the last year.
  - Last value of lipid control (cLDL) < 100 mg/dl.

**Indicator 10. Hypercholesterolemia: Cardiovascular Risk (CVR) record of hypercholesterolemia**

- Population included:
  - Between 35 and 74 years.
  - Diagnosis of hypercholesterolemia (ICD10 codes: E78%).
- Exclusion criteria:
  - Ischemic heart disease.
  - Cerebrovascular disease and/or transient ischemic attack.
  - Peripheral vascular disease.
- Compliance criteria:
  - To have at least one record of CVR calculation after the hypercholesterolemia diagnosis.

**Indicator 11. Atrial fibrillation (AF): Antiplatelet/anticoagulant treatment**

- Population included:
  - >14 years.
  - AF diagnosis (ICD10 codes: I48).
- Compliance criteria:
  - High risk:
    - No anticoagulant contraindication: At least one active treatment of Vitamin K antagonist.
    - Anticoagulant contraindication: At least one active treatment of Aspirin.
  - Moderate risk:
    - At least one active treatment of Vitamin K antagonist or Aspirin.

**Indicator 12. Arterial hypertension (AHT): Blood pressure (BP) control**

- Population included:
  - >14 years.
  - AHT diagnosis (ICD10 codes: I10%, I11%, I12%, I13%, I15%).
- Compliance criteria:
  - At least one blood pressure control in the last year.
  - BP control <150/95.

**Indicator 13. Arterial hypertension (AHT): Blood pressure (BP) control in at-risk population (IHD, DM, CVD/TIA, Chronic Kidney Failure)**

- Population included:
  - >14 years.
  - At least one of the following diagnoses:
    - IHD (ICD10 codes: I20%, I21%, I22%, I23%, I24%, I25%).
    - DM (ICD10 codes: E11%, E12%, E13%, E14%).
    - CVD/TIA (ICD10 codes: G45%, G46%, I63%, I64, I67.8, I67.9, I69, I69.3,I69.4,I69.8).
    - Chronic Kidney Failure (ICD10 codes: N18%, N19).
- Compliance criteria:
  - At least one blood pressure control in the last year.
  - BP control <140/90.

**Indicator 14. Heart failure (HF): ACEI/ARB treatment**

- Population included:
  - >14 years.
  - HF diagnosis (ICD10 codes: I50%, I11.0, I13.0, I13.2).
- Compliance criteria:
  - At least one active treatment of angiotensin-converting-enzyme inhibitor / angiotensin receptor blockers.

**Indicator 15. Heart failure (HF): Beta-blockers treatment**

- Population included:
  - >14 years.
  - HF diagnosis (ICD10 codes: I50%, I11.0, I13.0, I13.2).
- Exclusion criteria:
  - Atrioventricular block (second and third degree).
- Compliance criteria:
  - At least one active treatment of beta-blockers.

**Indicator 16. Chronic hepatitis C: Vaccination for hepatitis B virus**

- Population included:
  - Between 35 and 79 years.
  - Chronic hepatitis C diagnosis (ICD10 code B18.2)
- Exclusion criteria:
  - Hepatitis B diagnosis (ICD10 codes: B16, B18.0, B18.1).
- Compliance criteria:
  - 3 doses of hepatitis B vaccine.
  - Or at least one dose in the last 9 months.

**Indicator 17. Alcohol use: Screening for alcohol use**

- Population included:
  - Between 15 and 79 years.
- Compliance criteria:
  - At least one screening for alcohol use in the last two years.

**Indicator 18. Tobacco use: Screening for tobacco use in at-risk population**

- Population included:
  - >14 years.
  - At least one of the following diagnoses:
    - IHD (ICD10 codes: I20%, I21%, I22%, I23%, I24%, I25%).
    - DM (ICD10 codes: E11%, E12%, E13%, E14%).
    - Asthma (ICD10 codes: J45%, J46).
    - Chronic obstructive pulmonary disease (ICD10 codes: J42, J43%, J44%).
    - HF (ICD10 codes: I50%, I11.0, I13.0, I13.2).
    - AHT (ICD10 codes: I10%, I11%, I12%, I13%, I15%).
- Compliance criteria:
  - Screening for tobacco use.
  - A register of the condition of non smoker.

**Indicator 19. Tobacco use: Smoking cessation**

- Population included:
  - Between 15 and 79 years.
  - Smoking status at the beginning of the year (ICD10 codes F17%, T65.2 or ICS specific code of “Smoker”).
- Compliance criteria:
  - Non smoking status in the last year.

**Indicator 20. Diabetes mellitus 2 (DM2): Screening and onset prevention**

- Population included:
  - >14 years.
  - DM2 diagnosis (ICD10 codes: E11%, E12%, E13%, E14%).
- Compliance criteria:
  - To have an assessment of the feet (for the screening and onset prevention of the diabetic foot).

**Indicator 21. Diabetes mellitus 2 (DM2): HbA1c control**

- Population included:
  - Between 15 and 79 years.
  - DM2 diagnosis (ICD10 codes: E11%, E12%, E13%, E14%).
- Compliance criteria:
  - At least one HbA1c control in the last year.
  - HbA1c control <8%.

**Indicator 22. Diabetes mellitus 2 (DM2): Screening and prevention of diabetic retinopathy**

- Population included:
  - Between 15 and 79 years.
  - DM diagnosis (ICD10 codes: E11%, E12%, E13%, E14%).
- Compliance criteria:
  - To have a screening of diabetic retinopathy in the last 2 years.

**Indicator 23. Diabetes mellitus 2 (DM2): ACEI/ARB treatment in DM2 with chronic nephropathy**

- Population included:
  - >14 years.
  - DM diagnosis (ICD10 codes: E11%, E12%, E13%, E14%).
  - Chronic nephropathy diagnosis (ICD10 codes N18%, N19).
- Compliance criteria:
  - At least one active treatment of angiotensin-converting-enzyme inhibitor / angiotensin receptor blockers.

**Indicator 24. Cognitive deterioration: Syndrome diagnosis, new cases of cognitive deterioration**

- Population included:
  - >64 years.
  - New dementia diagnosis (ICD10 codes F00%, F01%, F02%, G30%).
- Compliance criteria:
  - To have some psychometric tests in primary care in the last year.

**Indicator 25. Cognitive deterioration: Home health care interventions for a safe living environment for patients with dementia**

- Population included:
  - >64 years.
  - New dementia diagnosis (ICD10 codes F00%, F01%, F02%, G30%).
  - Included in the Catalan program of Home health care.
- Compliance criteria:
  - To have an intervention about safe living environment at home.

**Indicator 26. Cognitive deterioration: Assessment of caregiver burnout**

- Population included:
  - >64 years.
  - New dementia diagnosis (ICD10 codes F00%, F01%, F02%, G30%).
  - Included in the Catalan program of Home health care.
  - With a caregiver identified.
- Compliance criteria:
  - To have an assessment of the caregiver burnout in the last year (using different tests such as Zarit test).

**Indicator 27. Impacted earwax: Removal of earwax plugs in primary care**

- Population included:
  - >14 years.
  - With an impacted earwax registered.
- Compliance criteria:
  - Removal of earwax plugs in primary care.

**Indicator 28. Asthma: Diagnosis of new cases of asthma**

- Population included:
  - >14 years.
  - New asthma diagnosis (ICD10 codes: J45%, J46).
- Compliance criteria:
  - To have a spirometry with bronchodilator test in the last 18 months.

**Indicator 29. Vaccinations: Flu shots in patients aged ≥60 years**

- Population included:
  - >59 years.
- Compliance criteria
  - A dose of influenza vaccine administered (between September 1st and December 31st).

**Indicator 30. Vaccinations: Flu shots in at-risk patients**

- Population included:
  - Between 15 and 59 years.
  - At least one of the following diagnoses:
    - IHD (ICD10 codes: I20%, I21%, I22%, I23%, I24%, I25%).
    - CVD/TIA (ICD10 codes: G45%, G46%, I63%, I64, I67.8, I67.9, I69, I69.3,I69.4,I69.8).
    - DM2 (ICD10 codes: E11%, E12%, E13%, E14%).
    - DM1 (ICD10 codes: E10%).
    - HF (ICD10 codes: I50%, I11.0, I13.0, I13.2).
    - Chronic Kidney Failure (ICD10 codes: N18%, N19).
    - Valvular heart disease (ICD10 codes: I05%, I06%, I07%, I08%, I34%, I35%, I36%, I37%, Q22%, Q23%).
    - Asthma (ICD10 codes: J45%, J46).
    - Chronic obstructive pulmonary disease (ICD10 codes: J42, J43%, J44%).
    - HIV (ICD10 codes: B20%, B21%, B22%, B23%, B24).
- Compliance criteria
  - A dose of influenza vaccine administered (between September 1st and December 31st).

**Indicator 31. Vaccinations: Pneumonia shots in patients aged ≥60 years**

- Population included:
  - >59 years.
- Compliance criteria
  - A dose of pneumococcal vaccine.

**Indicator 32. Vaccinations: Tetanus shots**

- Population included:
  - >39 years.
- Compliance criteria:
  - A dose of tetanus vaccine in the last 25 years.

**Indicator 33. Chronic obstructive pulmonary disease (COPD): Diagnosis of new cases**

- Population included:
  - >39 years.
  - A new COPD diagnosis (ICD10 codes: J42, J43%, J44%).
- Compliance criteria:
  - To have a spirometry in the last 18 months.

**Indicator 34. Chronic obstructive pulmonary disease (COPD): Training in use of COPD inhalers**

- Population included:
  - >39 years.
  - COPD diagnosis (ICD10 codes: J42, J43%, J44%).
- Compliance criteria:
  - To have registered a training in use of COPD inhalers in the last year.

**Indicator 35. Home health care (ATDOM): Case complexity assessment of patients with ATDOM**

- Population included:
  - >14 years
  - Included in the Catalan program of Home health care (ATDOM).
- Exclusion:
  - Dementia diagnosis (ICD10 codes F00%, F01%, F02%, G30%). Exclusion for cognitive assessment.
- Compliance criteria:
  - To have ​​the assessment of dependence, cognitive state and social risk at least once in the last year. We use different tests such as Barthel test and Pfeiffer test.

**Indicator 36. Home health care (ATDOM): Assessment of pressure ulcers risk**

- Population included:
  - >14 years
  - Included in the Catalan program of Home health care (ATDOM).
- Exclusion:
  - Decubitus ulcers (ICD10 codes: L89).
  - Or skin lesion.
- Compliance criteria:
  - To have an assessment of pressure ulcers risk (Braden test) at least once in the last year.

**Indicator 37. PREALT (Primary Care visit after hospital discharge): Contact within 48 hours with PREALT patients**

- Population included:
  - >14 years.
  - Patients referred to primary care after hospital discharge.
- Compliance criteria:
  - Primary care contact within 48 hours.

**Indicator 38. Nephritic colic: Proper treatment of nephritic colic**

- Population included:
  - >14 years
  - New nephritic colic diagnosis (ICD10 codes: N20%, N21%, N23).
- Compliance criteria:
  - To not have treatment with hyoscine butylbromide the week prior and after the diagnosis.

**Indicator 39. Prostate cancer: Avoid improper use of PSA**

- Population included:
  - Men >74 years.
- Exclusion:
  - Prostate cancer or benign prostatic hyperplasia diagnosis (ICD10 codes: C61, D07.5, D29.1, D40.0, N40).
- Compliance criteria:
  - To not request a PSA test.

**Clinical Condition in Children**

**Indicator 40. Preventive care: Screening for congenital metabolic diseases**

- Population included:
  - Between 6 and 18 months.
- Compliance criteria:
  - Screening for congenital metabolic diseases (phenylketonuria, hypothyroidism, cystic fibrosis) before 6 months old.

**Indicator 41. Preventive care: Introduction of foods at recommended stages**

- Population included:
  - Between 6 and 18 months.
- Compliance criteria:
  - To have the introduction of foods at recommended stages:
    - Solid food from 4 months.
    - Gluten from 7 months.

**Indicator 42. Preventive care: Systematic infant vaccinations (0-14 years)**

- Population included:
  - <15 years.
- Compliance criteria:
  - The number of doses required depending on the age according to routine vaccination schedule of Catalonia [Departament de Salut. *Diari Oficial de la Generalitat de Catalunya*. DOGC 5257. 2008; available on: http://www.gencat.cat/diari/5257/08310104.htm].

**Indicator 43. Preventive care: Control of growth and development (0-2 years)**

- Population included:
  - Between 0 and 2 years
- Compliance criteria:
  - Between 0 and 1 year
    - At least 3 controls of growth (weight, height, cranial perimeter) and psychomotor development (postural, manipulation, language and sociability).
  - Between 1 and 2 years
    - At least 1 control of growth (weight, height, cranial perimeter) and psychomotor development (postural, manipulation, language and sociability).

**Indicator 44. Preventive care: Screening for passive smoking ≤2-year-olds**

- Population included:
  - Between 0 and 2 years
- Compliance criteria:
  - Smoking screening and anti-smoking assessment to their parents.

**Indicator 45. Preventive care: Maintenance of maternal lactation**

- Population included:
  - Between 3 and 15 months
- Compliance criteria:
  - Maintenance of maternal lactation at least the first three months of life.

**Indicator 46. Preventive care: Measles vaccination at 13 years**

- Population included:
  - <= 13 years
- To have natural immunity or one dose of measles vaccine.

**Indicator 47. Preventive care: Dental cavity preventive treatment (6-12 years)**

- Population included:
  - Between 6 and 12 years.
  - High risk of dental cavity.
- Compliance criteria:
  - To have preventive treatment (Fluoride therapy).

**Indicator 48. Preventive care: Screening for ocular diseases (0-6 years)**

- Population included:
  - Between 0 and 6 years.
- Compliance criteria:
  - Screening for ocular diseases (strabismus and visual acuity) at least once.

**Indicator 49. Preventive care: Flu shots in at-risk children <15 years**

- Population included:
  - <15 years
  - At least one of the following diagnoses:
    - HF (ICD10 codes: I50%, I11.0, I13.0, I13.2).
    - Valvular heart disease (ICD10 codes: I05%, I06%, I07%, I08%, I34%, I35%, I36%, I37%, Q22%, Q23%).
    - Asthma (ICD10 codes: J45%, J46).
    - Chronic obstructive pulmonary disease (ICD10 codes: J42, J43%, J44%).
    - Chronic Kidney Failure (ICD10 codes: N18%, N19).
    - HIV (ICD10 codes: B20%, B21%, B22%, B23%, B24).
- Compliance criteria:
  - A dose of influenza vaccine administered (between September 1st and December 31st).

**Indicator 50. Preventive care: Screening for toxic habits (11-14 years)**

- Population included:
  - Between 11 and 14 years
- At least one screening for toxic habits (tobacco, alcohol and other drugs).

**Indicator 51. Increasing capacity to resolve cases at primary care level: Umbilical hernia in children ≤3 years**

- Population included:
  - Between 0 and 3 years
  - Umbilical hernia diagnosis (ICD10 codes: K42, K42.9).
- Compliance criteria:
  - To resolve the case at primary care level (to not send to surgery service).

**Indicator 52. Increasing capacity to resolve cases at primary care level: Contagious mollusk and viral warts (0-14 years )**

- Population included:
  - <15 years
  - contagious mollusk and viral warts diagnosis (ICD10 codes: B07, B08.1).
- Compliance criteria:
  - To resolve the case at primary care level (to not send to dermatology service).

**Indicator 53. Increasing capacity to resolve cases at primary care level: Neonatal dacryocystitis <9 months**

- Population included:
  - <9 months
  - Neonatal dacryocistitis (ICD10 codes: P39.1).
- Compliance criteria:
  - To resolve the case at primary care level (to not send to ophthalmology service).

**Indicator 54. Acute disease: Treatment of acute gastroenteritis (3 months to 14 years)**

- Population included:
  - Between 3 months and 14 years.
  - Infectious gastroenteritis diagnosis (ICD10 codes: A09).
- Exclusion criteria:
  - Immunosuppression condition (ICD10 codes: D80%, D81%, D82%, D83%, D84%).
  - Drepanocytosi (ICD 10 codes: D57%).
- Compliance criteria:
  - To not have prescription of antibacterial drugs or antidiarrhoeal within 72 hours after the diagnosis.

**Indicator 55. Acute disease: Treatment of tonsillitis, pharyngitis or pharyngotonsillitis <3 years**

- Population included:
  - < 3 years.
  - Tonsillitis, pharyngitis or pharyngotonsillitis diagnosis (ICD10 codes: J02, J02.9, J03, J03.9).
- Compliance criteria
  - To not have prescription of antibacterial drugs within 72 hours after the diagnosis.

**Indicator 56. Acute disease: Treatment of acute bronchiolitis <2 years**

- Population included:
  - <2 years
  - New bronchiolitis or bronchitis diagnosis (ICD10 codes: J20, J20.9, J21, J21.9).
- Compliance criteria:
  - To not have prescription of antibacterial drugs within 72 hours after the diagnosis.

**Indicator 57. Acute disease: Treatment of catarrh in upper respiratory infection or flu <15 years**

- Population included:
  - <15 years
  - Catarrh in upper respiratory infection or flu diagnosis (ICD10 codes: J00, J11).
- Compliance criteria:
  - To not have prescription of antibacterial drugs within 72 hours after the diagnosis.

**Indicator 58. Acute disease: Treatment of acute nonsuppurative otitis media (2-14 years )**

- Population included:
  - Between 2 and 14 years.
  - Acute nonsuppurative otitis media diagnosis (ICD10 codes: H65, H65.0, H65.1, H65.9, H66, H66.4, H66.9).
- Compliance criteria:
  - To not have prescription of antibacterial drugs within 48 hours after the diagnosis.

**Indicator 59. Chronic disease: Diagnosis of childhood asthma (7 a 14 years)**

- Population included:
  - Between 7 and 14 years.
  - New asthma diagnosis (J45%).
- Compliance criteria:
  - To have a spirometry with bronchodilator test in the last 18 months.

**Indicator 60. Chronic disease: Calculation of body mass index in obesity or weight gain (6-14 years )**

- Population included:
  - Between 6 and 14 years
  - Obesity diagnosis or weight gain (ICD10 codes: E66, E66.0, E66.1, E66.2,E66.8, E66.9, R63.5).
- Compliance criteria:
  - To calculate the body mass index.

**Indicator 61. Social assessment: Social assessment of children with a disability (<15 years)**

- Population included:
  - < 15 years.
  - At least one of the following diagnoses:
    - To have registered the ICS specific code of “Children with a disability”.
    - Cerebral palsy (ICD 10 codes: G80, G80.0, G80.3, G80.4, G80.9).
    - Down syndrome (ICD10 codes: Q90, Q90.9).
    - Blindness (ICD10 codes: H54.0).
    - Deafness (ICD10 codes: H91.3).
    - Autism (ICD10 codes: F84.0, F84.1).
- Compliance criteria:
  - To have a social assessment.
